# Genome organization and the role of centromeres in evolution of the erythroleukaemia cell line HEL

**DOI:** 10.1093/emph/eot020

**Published:** 2013-10-01

**Authors:** Ruth N. MacKinnon, Meaghan Wall, Adrian Zordan, Srilakshmi Nutalapati, Bruce Mercer, Joanne Peverall, Lynda J. Campbell

**Affiliations:** ^1^Victorian Cancer Cytogenetics Service, St Vincent’s Hospital, Melbourne, Fitzroy, Vic, Australia; ^2^Department of Medicine, St Vincent’s Hospital, University of Melbourne, Melbourne, Vic, Australia; and ^3^PathWest Department of Diagnostic Genomics, QEII Medical Centre, Nedlands, WA, Australia

**Keywords:** leukaemia, cell line, chromosome, centromere, HEL, SNP array

## Abstract

**Background and objectives:** The human erythroleukaemia (HEL) cell line has a highly rearranged genome. We matched whole chromosome analysis with cytogenomic microarray data to build a detailed description of these rearrangements.

**Methodology:** We used a combination of single nucleotide polymorphism array and multiple fluorescence *in situ* hybridization approaches, and compared our array data with publicly available data for different sublines of HEL. B allele frequencies revealed the fate of each homologue for most chromosomes.

**Results:** At least two instances of the breakage–fusion–bridge cycle appear to have facilitated amplification of oncogenes and deletion of tumour suppressor genes. Because our study included centromere identification, we found that some abnormal chromosomes had centromeres that did not match the identity of the rest of the chromosome.

**Conclusions and implications:** This study highlights the variety of complementary methods required to understand remodelling of the genome in cancer and uncover some of the mechanisms involved. We present evidence of centromere capture as a means of preserving broken chromosome segments. Testing for another highly repetitive DNA region, the nucleolus organizer region, helped identify the steps involved in chromosome 9 copy number aberrations. Increased use of techniques for identifying centromeres and other repetitive DNA regions will add to our understanding of genome remodelling and evolution. The pattern of chromosome 20 aberration in HEL supports an association of 20q11.21 amplification with erythroleukaemia (acute myeloid leukaemia subtype M6) in the context of 20q12 deletion. The differences between the karyotypes in different HEL sublines highlight the constantly evolving genomes of cultured cell lines.

## Abstract (precis)

We describe in detail the abnormal chromosomes of HEL, a human leukaemia cell line grown in many laboratories. Complementary high resolution and whole chromosome analysis tools, including the localisation of repetitive DNA sequences on intact chromosomes, are essential for understanding genome remodelling and evolution in cancer cells.

## INTRODUCTION

Accurate characterization of the genome aberrations of a cell line is important for its use as a research tool. The structure and organization of abnormal chromosomes can provide clues as to how the genome rearrangements were derived, and therefore what genetic errors have promoted oncogenicity. Sequence data are available for the human erythroleukaemia (HEL) cell line [1], but the complex genome rearrangements have not been well described. We have made a comprehensive characterization of the abnormal chromosomes in HEL. The use of fluorescence *in situ* hybridization (FISH) to study intact metaphase chromosomes has helped identify translocation partners and centromere content, details which are not readily obtained by other methods.

The HEL cell line was established from a patient with a therapy-related acute myeloid leukaemia (AML), subtype erythroleukaemia, following treatment for Hodgkin lymphoma [2]. It is capable of globin synthesis [2], and is widely used to study cell biology and differentiation. There was limited karyotypic information at the time this cell line was established. Two Y chromosomes and two double-minute chromosomes were reported. The malignant blasts exhibited a triploid karyotype similar to the established cell line, indicating that triploidization had occurred *in vivo* [2].

The most detailed published karyotypes were determined for HEL [3, 4] with the aid of whole chromosome painting probes, and for the HEL-contaminated Dami cell line [5]. Results of single nucleotide polymorphism (SNP) array analysis are available at the Cancer Genome Project (CGP) website [6]. The Cancer Cell Line Encyclopedia (CCLE) reports several modes of characterization, including OncoMap sequencing of cancer-relevant genes and SNP array, for two sources of HEL: the German Collection of Microorganisms and Cell Cultures (DSMZ, Braunschweig, Germany) and the American Tissue Culture Collection (HEL 92.1.7) (ATCC, Manassas, VA) [1].

Using a combination of multicolour FISH (M-FISH), multicolour banding (M-BAND), targeted FISH and SNP array, we have made a comprehensive characterization of the chromosome rearrangements in a sub-population of the HEL cell line. Another aim of this study was to further elucidate the chromosome 20 abnormalities highlighted by MacGrogan *et al.* [7] in this cell line. Deletion of a hypothetical, as yet unconfirmed, tumour suppressor gene at 20q12 is a non-random event in myeloid malignancies [8–11]. MacGrogan *et al.* [7] tested for loss of the common deleted region (CDR) at 20q12, and identified loss of heterozygosity (LOH) at 20q12, in HEL and a number of other cell lines. Their assumption was that loss of the critical tumour suppressor gene from this region contributed to the oncogenic phenotype of these cell lines. Here, we show that 20q deletion comprises one of many known myeloid malignancy copy number aberrations (CNAs) that have occurred in HEL.

## MATERIALS AND METHODS

The cell line HEL was cultured in RPMI 1640 containing 10% foetal bovine serum, glutamine, penicillin and streptomycin in air containing 5% CO_2_ and harvested to produce metaphase chromosomes using standard procedures [12]. Chromosomes were G-banded using Leishman stain according to standard techniques [13].

FISH was carried out on metaphase chromosomes prepared according to standard techniques using the Vysis (Abbott Molecular Inc., Downers Grove, IL) co-denaturation protocol. FISH was analysed using a Zeiss Axioplan 2 fluorescence microscope (Carl Zeiss, Jena, Germany), and captured with an Isis capturing and analysis station (Metasystems, Altlussheim, Germany). FISH probes were used both to localize rearranged segments identified by SNP array to abnormal chromosomes and as markers to help identify known abnormal chromosomes. M-BAND was used to localize rearranged segments and to help identify the breakpoints of balanced translocations.

Bacterial artificial chromosomes (BACs) were selected on the basis of their map positions in the Ensembl database (www.ensembl.org) and labelled with SpectrumOrange and/or SpectrumGreen (Abbott Molecular Inc.) to generate locus-specific FISH probes mapping to the regions of interest. All BACs were checked for chromosomal location before use, and were hybridized at a final concentration of 10–15 ng/µl.

The following commercial FISH probes were used: Vysis probes LSI D20S108 (20q12) SpectrumOrange for the *D20S108* locus from the CDR at 20q12 [8], LSI MLL dual colour, break apart probe (11q23), LSI 19p13 SpectrumGreen/LSI 19q13 SpectrumOrange, LSI PML/RARA dual fusion probe (Abbott Molecular Inc.), Poseidon *MECOM* probe EVI t(3;3); inv(3)(3q26) dual colour (Kreatech, Amsterdam) and centromere-specific probes (CEP4 SpectrumOrange, CEP9 SpectrumOrange, CEP10 SpectrumGreen, CEP11 (D11Z1) SpectrumOrange, CEP17 (D17Z1) SpectrumAqua, CEP18 (D18Z1) SpectrumOrange, CEP20 (D20Z1) SpectrumOrange (Abbott Molecular Inc.); Poseidon centromere probes SE1/5/19 (Green) and SE20 D20Z1 (aqua) (Kreatech, Amsterdam); and Aquarius MYB deletion Probe which contains probes for *MYB* (6q23) and the 6 centromere (*CEP6*) (Cytocell, Cambridge). The Aquarius LPE NOR probe (Cytocell) specifically identifies the nucleolar organizer region (NOR) on the acrocentric chromosome short arms. Combinations of two or three probes labelled with contrasting fluorochromes were hybridized to metaphases using the Vysis co-denaturation protocol. Co-denaturation was at 72°C for 2 min. When combining Poseidon probes with Vysis or BAC probes, the amount of Poseidon probe used (not diluted with the buffer provided) was one-tenth to one-fifth of the final volume, the recommended Poseidon pre-treatment protocol was used, and probes and chromosomes were co-denatured at 72.5°C for 3 min.

M-FISH and M-BAND were carried out using Metasystems (Metasystems, Altlussheim, Germany) M-FISH (XCyte 24) and M-BAND (XCyte 3, 4, 10, 11, 13, 15, 18, 19 and 20) probes according to the manufacturer’s instructions.

Signal intensity was estimated for single locus probes by comparing the signal on the abnormal chromosome to the signal on the normal chromosome. The number and relative intensity of signals per cell were assessed blind and averaged over at least ten metaphases.

SNP array was carried out on the Illumina CytoSNP 12 platform (Illumina, San Diego, CA), with DNA extracted from cultured cell lines in the log phase of growth using a DNeasy Cell and Tissue kit (Qiagen, Germantown, MD). Data were analysed using KaryoStudio software (Version 1.2, Illumina). SNP array data have been deposited at the Genome Expression Omnibus (GEO) with accession number GSE41964.

Chromosome composition and breakpoints were determined using the G-band, M-FISH and M-BAND images, SNP array data, and single locus FISH data when available. The breakpoints of CNAs and unbalanced translocations were determined from SNP array data showing changes in copy number and B allele frequencies (BAFs). In addition, the breakpoints of whole arm translocations were determined by centromere probes when available. Karyotypes are written according to the ISCN (2013) [14].

The NOR probe helped identify the sites of translocation in apparent whole arm translocations involving the acrocentric chromosomes (13, 14, 15, 21 and 22). Balanced translocations were identified by M-FISH, and their breakpoints were determined by M-BAND.

SNP array results were compared with SNP array data available online using the cytogenetic visualization tools available for these data: the CGP (visualized with the CGH Viewer using the PICNIC algorithm) and CCLE (using the Integrated Genome Viewer). CCLE data were also downloaded from GEO (HEL92.1.7, ATCC accession number GSM888146; HEL, DSMZ accession number GSM888147) and visualized with the Affymetrix ChAS software (version 1). This allowed direct comparison of copy number and heterozygosity with our data for each chromosome region.

## RESULTS AND DISCUSSION

Combining FISH, M-FISH and M-BAND results with CNA and BAF information provided by SNP array allowed us to determine the content and structure of most of the abnormal chromosomes produced by the complex genome reorganization in the HEL cell line.

**Figure 1.**
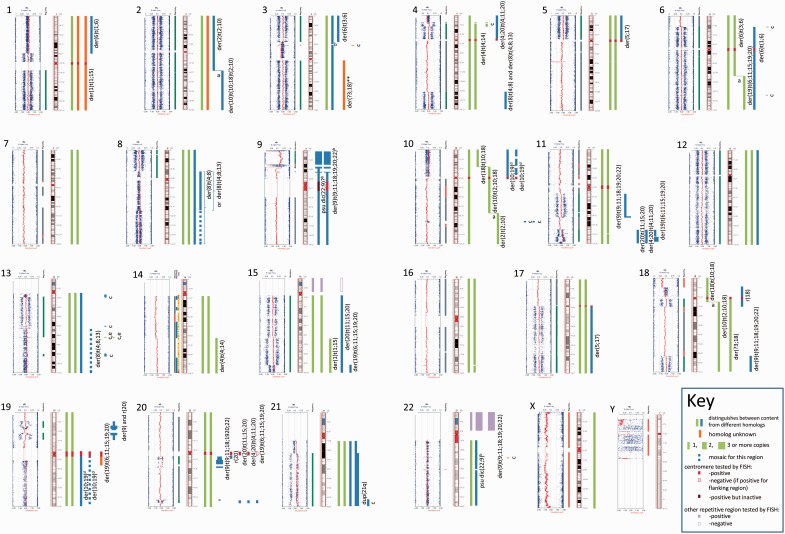
SNP array images for each chromosome with their interpretation. SNP array results are arranged by chromosome. For each chromosome, the output from the KaryoStudio software is displayed against the idiogram. Vertical bars aligned to the right of the idiograms define each segment of the chromosome and its location in a normal or abnormal chromosome. The abnormal chromosomes containing each region are identified when possible. Chromosomes derived from the same homologue are represented in bars of the same colour. Parental inheritance cannot be inferred for different chromosomes represented by the same coloured vertical bar. Heterochromatic regions are assumed to be present across SNP array gaps; however, when breakpoints were adjacent to these regions, presence or absence of the heterochromatic regions (4, 5, 10, 17, 19 and 20 centromeres and nucleolus organizer region) is shown by a solid bar (present) or empty box (absent) as shown in the key, if tested by FISH. (**a**) Horizontal line, balanced translocation; (**b**) it is not known which homologue has this single copy deletion; (**c**) the abnormal chromosome containing this segment was not located; (**d**) slight splitting of the AB column suggests that there is a low level of mosaicism for the second copy of this chromosome; (**e**) CGP data confirm that the additional 2–3 copies of 13q21.33 were derived from the normal homologue

**Figure 2.**
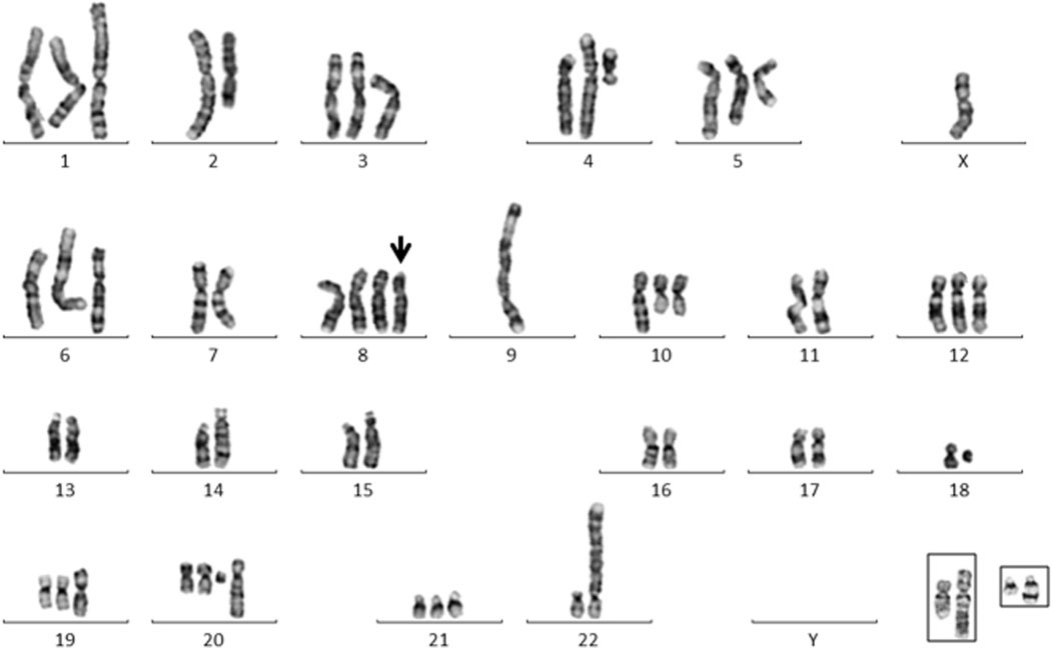
A representative HEL G-banded karyotype with the der(8)t(4;8;13) (arrow). The karyotype is missing a chromosome 13, the der(10)t(2;10;18), and the dup(21). The der(10) (left) and dup(21) are shown in insets, each paired with a normal homologue

**Figure 3.**
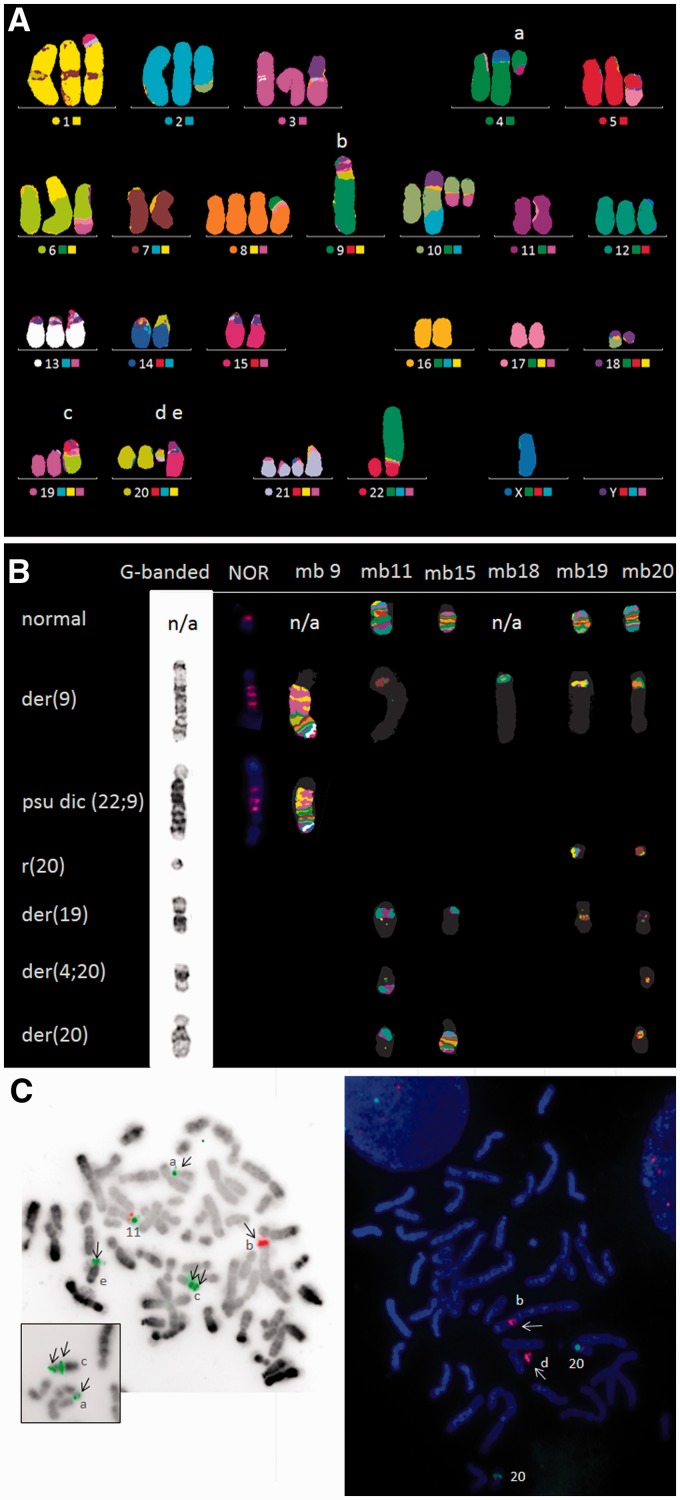
Representative FISH images showing (**a**) the der(4;20), (**b**) der(9), (**c**) der(19), (**d**) r(20), (**e**) der(20). (**A**) M-FISH of a metaphase with the der(8)t(4;8). The coloured circles to the left of each chromosome name or number show the false colour representing each normal chromosome. (**B**) G-banded, NOR and M-BAND (mb) images (left to right) of the normal homologues and abnormal chromosomes involving chromosomes 9 and 20 (n/a, not applicable as no normal chromosome 9 or 18 was available; the normal NOR pattern is shown on an F group chromosome). NOR staining shows 3–4 signals in the der(9) and psu dic(22;9); M-BAND confirms the presence of sequences from chromosomes 9, 11, 18, 19 and 20 in the der(9); 19 and 20 in the r(20); 11, 15, 19 and 20 in the der(19); 11 and 20 in the der(4;20); and 11, 15 and 20 in the der(20) (M-BAND for chromosomes 4, 6 and 22 not shown). (**C**) BAC FISH images showing the location of short sections of chromosomes 11 and 20. Left, metaphase confirming duplication of RP11-676M06 (11q24.2–3, green) in the der(19); and amplification of RP11-418A08 at the site of translocation with chromosome 18 in the der(9) (11q21–q22.1, red) (false colour image). Inset, another example showing duplication of RP11-676M06 (11q24.2–3). Right, metaphase demonstrating two copies of RP11-69I10 at 20q12 (green) and amplification of RP11-483M19 (20q11.21, red) in the ring(20) and der(9). Arrows denote the signal on the abnormal chromosomes

The SNP array KaryoStudio images for each chromosome are presented in Fig. 1. G-band, M-FISH, M-BAND and FISH results are presented in Figs 2 and 3 and Tables S1–S4.

The SNP array BAF measures the relative ratios of the paternally inherited homologue and the maternally inherited homologue. Thus, analysis of the copy number plots and the BAF plots allowed us to assign the sections of each chromosome that are defined by different BAF values to a particular chromosome derivative. These sections are shown in Fig. 1 aligned with each chromosome idiogram. For most chromosomes, the BAFs also allowed us to determine which of the two homologues was involved in the abnormal chromosomes (Fig. 1). The un-rearranged homologue was duplicated in most instances (Fig. 1). The position and order of these chromosome segments within the abnormal chromosomes were determined by M-FISH, with the help of M-BAND and single locus FISH when necessary.

Terminal segments were assigned to the telomeric end of an abnormal chromosome. The duplicated :11q23.2->11qter segment was assigned to the two derivatives that were shown by M-FISH to have chromosome 11 material at the end of a chromosome arm. The non-telomere-containing :11q23.2->11q25: segment was assumed to form part of an inverted duplication :11q23.2-> 11q25::11q25->11q24.3: and assigned to the der(19) (der(19) describes an abnormal derivative chromosome with a 19 centromere, although it may contain segments from other chromosomes), because M-FISH showed that the chromosome 11-positive section of the der(19) was flanked by chromosome 19 material at both ends. This interpretation was confirmed by FISH (Fig. 3B and C, Table S1).

The near-triploid karyotype determined by combining the results of these procedures is

56∼62,<3n>,X,-X,-Y[11],der(1)t(1;15)(p36.3;q22.31)[11],der(2)t(2;10)(q22;q24)[10],der(?3;18)(q1?0;q10)[10],der(4)t(4;14)(p15.33;q23.3)del(4)(p15.32p15.32)[11],der(4;20)(4pter->4p10::20q10->20q11.1::20q13.33->20q13.33::11q23.2->11qter)[10],der(5;17)(p10;q10)[10],der(6)t(1;6)(p21.3;p22.3)[11],der(6)t(3;6)(p14.3;q21)[11],-7[11],+der(8)t(4;8)(q32.1;p12)[5],+der(8)(4qter->4q32.1::8p12->8q22.1::13q21.31->13qter)[6],-9[11],der(9)(18qter->18q22.1::11q22.1->11q13.1::20q13.33->20q13.33::20q11.21->20q11.23::20q11.23->20q11.22::20q11.22->20q11.23::20q11.23->20q11.21::19p13.?->19p13.?::22p11∼2->22p12∼3::9p24.3->9p21.3::9p21.3->9p21.2::9p21.1->9qter)[11],der(10)t(10;18)(p12;q11.2)t(2;10)(q22;q24)[11],der(10;19)(p10;q10)[11],+der(10;19)[9],-11[11],-14[11],-15[11],-16[11],-18[4],r(18)(::p11.23->q12::)[7],der(18)t(10;18)(p12.1;q11.2)[11],+der(19)(15qter->15q26.2::11q25->11q24.1::20q13.33->20q13.33::11q23.2->11q25::20q11.1->20q11.1::19q10->19q12::6q21->6qter)[11],+19[2],der(20)(15qter->15q11.2::20q10->20q11.1::20q13.33->20q13.33::11q23.2->11qter)[10],+r(20)(::20p11.1q11.21::19p13.?->19p13.?::)[10],dup(21)(q21.1qter)[10],psu dic(22;9)(p12∼3;p21.3)del(9)(p21.3p21.3)amp(9)(p24.3p21.2)amp(22)(p11p11∼3)[11],-22[11]

### Whole arm translocations

SNP array identified a number of whole arm translocations, in which the breakpoint occurred between the euchromatic regions of the two chromosome arms. Because SNP arrays do not identify where these breakpoints occur in relation to the centromere, we investigated this with FISH.

Using centromere probes for chromosomes 4, 5, 10, 17, 19 and 20, the following translocations were shown to contain centromeres from both chromosomes (Fig. 1 and Table S4):

- der(4;20)(4pter->4p10::20q10->20q11.1::20q13.33 ->20q13.33::11q23.2 ->11qter).
- der(5;17)(p10;q10).
- der(10;19)(p10;q10).

(der(4;20) describes a chromosome containing chromosomes 4 and 20 centromeres.) (Chromosomes 3 and 15 centromeres were not tested.)

### Recurrent AML aberrations

CNAs in HEL that are well-recognized in AML include deletion or LOH of 4q (*TET2*) [15], a der(5;17)(p10;q10) (5q and 17p (*TP53*) deletion) [16], loss of chromosome 7 [17], gain of 8q22 [18], gain of *MLL* (11q23) [19, 20], 20q12 deletion and amplification of 21q (*RUNX1*) [21]. There was partial deletion of *FHIT* at the 5′ end (3p14). *FHIT* aberration has only rarely been noted in AML [22, 23]. Interestingly, gain of the fifth copy of *MLL* and the fourth copy of 8q22, and LOH of 4q in the DSMZ and ATCC specimens but not the CGP specimen, suggests that these aberrations have been acquired *in vitro*. These may be examples of selective pressure *in vitro* resembling selective pressure *in vivo.*

### Abnormalities of chromosomes 9 and 20

The chromosome abnormalities involving chromosomes 9 and 20 in HEL appeared to be stable, functionally monocentric chromosomes, and their stability is supported by the overall similarity between sublines. The content and organization of these chromosomes pointed to the mechanisms by which they were produced. Breakage–fusion–bridge (BFB) cycles induced by the formation of dicentric chromosomes were the likely cause of CNAs involving these chromosomes, as described below.

#### Chromosome 9

**Figure 4.**
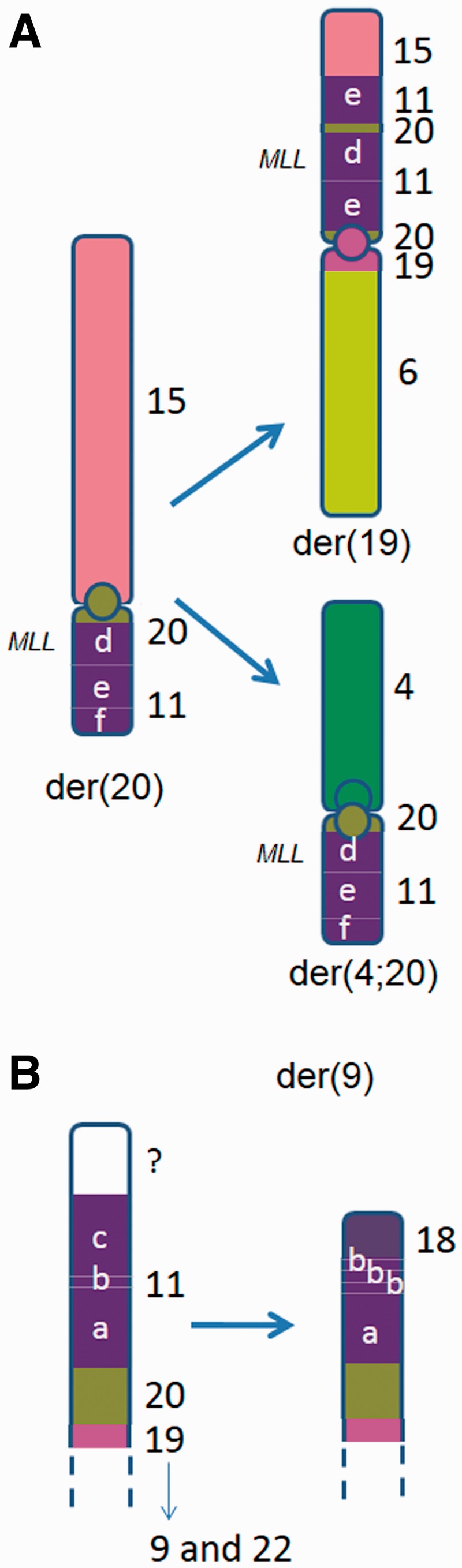
Schematic diagrams showing the derivation of (**A**) the der(19) and der(4;20) that have evolved from the der(20). CNAs and LOH associated with the der(4;20) were not present in the CGP specimen, evidence that it arose in the ATCC and DSMZ common ancestor after separation from the CGP line. *MLL* was present in the chromosome 11 segment labelled ‘d’; (**B**) the telomeric end of the der(9) unique to the VCCS HEL specimen (the chromosome 9 and 22 elements are not shown). The amplified segment of 11q23 containing *MLL* is represented by ‘e’

**Figure 5.**
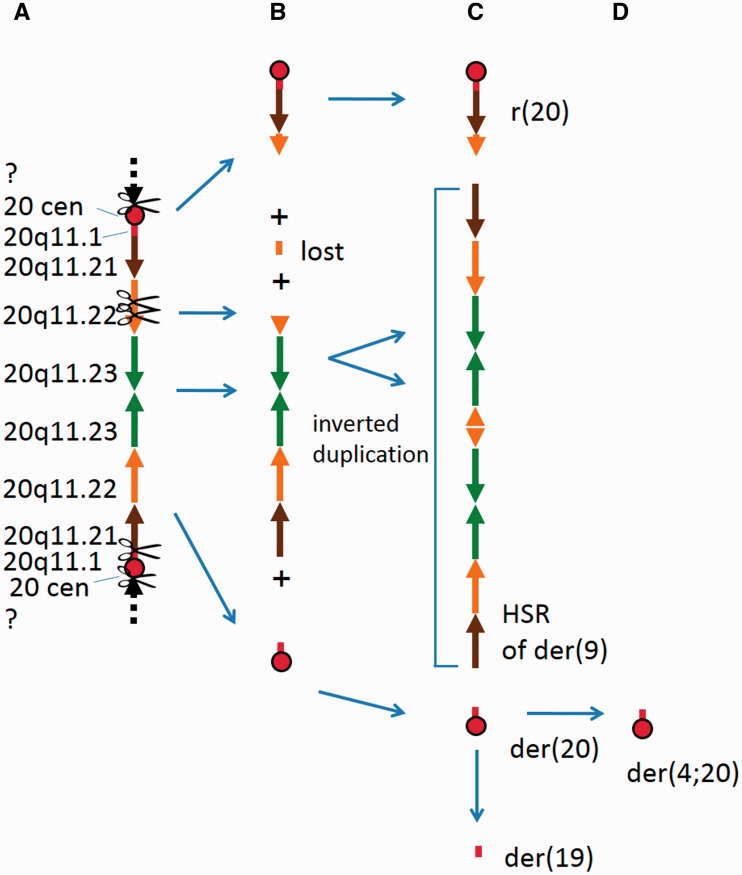
Hypothetical pattern of rearrangements producing the 20q11.2 aberrations in HEL. (**A**) ancestral isodicentric section flanked by two 20 centromeres (cen, centromere; ?, dashed lines represent surrounding material, content unknown); (**B**) hypothetical intermediate stage: sections derived by breakage; (**C**) the distribution of 20q11.2 elements in the abnormal chromosomes in HEL excluding the der(4;20); (**D**) the der(4;20) that arose *in vitro* from the der(20)

All regions of chromosome 9 were homozygous and there was no normal chromosome 9. A combination of M-FISH, M-BAND, chromosome 9, 11 and 20 single locus probes, and probes for the 9 centromere and NOR DNA repeats helped unravel the complex organization of these chromosomes (see karyotype, Figs 3–5). There was amplification of 9p24.3->9p21.2, 11q21/q22.1, 19p13 and 22p on the der(9) and amplification of 9p24.3->9p21.2 and 22p on the pseudodicentric derivative, psu dic(22;9) (see Figs 1 and 4B).

**Figure 6.**
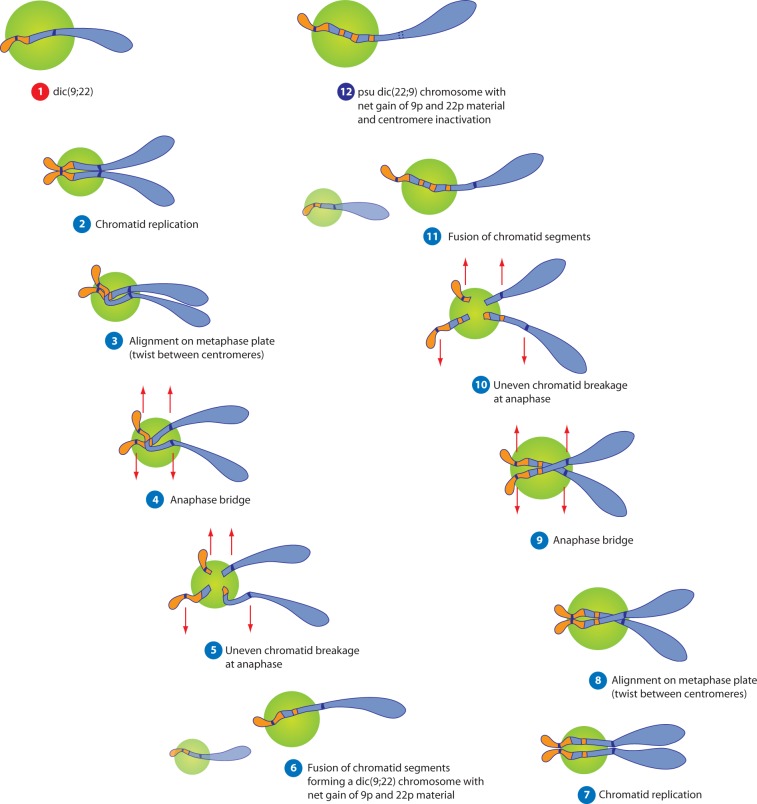
Schematic diagram showing how the psu dic(22;9) may have been formed from a dic(9;22) by the breakage-fusion-bridge cycle

Both the der(9) and psu dic(22;9) had similar patterns of alternating 9p and NOR-positive material (Fig. 3B). Positivity for the NOR probe in both derivatives showed that the common precursor was probably a dicentric chromosome, dic(9;22)(p24.3;p12∼3), containing NOR material from 22p. Amplification of material from only one arm of each chromosome, 9p and 22p, is typical of BFB-derived amplification between the centromeres of a dicentric chromosome. BFB CNAs arising in dicentric chromosomes occur between the two centromeres, i.e. only on one chromosome arm (see Fig. 6). This suggests the likely sequence of events as follows:

- Formation of an unstable dic(9;22) was followed by BFB cycles between the centromeres, producing CNA of 9p and 22p [24].
- This derivative(9;22) was duplicated.
- One copy of the der(9;22) was stabilized by inactivation of the 9 centromere to form the pseudodicentric (22;9).
- The other copy was stabilized by replacement of the chromosome 22 centromere and long arm with material from chromosomes 11, 18, 19 and 20 to form the der(9).

This process produced amplification (eight copies) of the homozygous V617F mutant *JAK2* allele [25, 26] and homozygous deletion of *CDKN2A*. *JAK2* mutation and homozygous deletion of *CDKN2A* are uncommon in AML [27–29]. However, homozygous *CDKN2A* deletion is often acquired during the immortalization of cell lines [30].

#### Chromosome 20

Two normal copies of one chromosome 20 homologue and five abnormal chromosomes were positive for the chromosome 20 M-BAND probe (Fig. 3B). The five abnormal chromosomes had been derived from the one chromosome 20 homologue. Both SNP array (Fig. 1) and FISH (Table S2) showed that none of these contained the 20q12 CDR [8], confirming LOH as reported previously [7].

M-BAND, BAC FISH and centromere FISH helped us to determine the distribution of the different sections of the rearranged chromosome 20 homologue (Table S2, Fig. 3B and C). Note that the XCyte 20 M-BAND probe could detect the paracentric chromosome 20 region on the der(4;20), der(19) and der(20) that is not represented on the SNP array (Table S2, Fig. 5).

The chromosome 20 abnormalities were

- der(4;20)(4pter->4p10::20q10->20q11.1::20q13.33 ->20q13.33::11q23.2 ->11qter).
- der(9)(18qter->18q22.1::11q22.1 ->11q13.1::20q13.33 ->20q13.33::20q11.21 ->20q11.23::20q11.23 ->20q11.22::20q11.22 ->20q11.23::20q11.23 ->20q11.21::19p13.?->19p13.?::22p11∼2 ->22p12∼3::9p24.3 ->9p21.3::9p21.3 ->9p21.2::9p21.1 ->9qter).
- der(19)(15qter->15q26.2::11q25->11q24.1::20q13.33 ->20q13.33::11q23.2 ->11q25::20q11.1 ->20q11.1::19q10->19q12::6q21->6qter).
- der(20)(15qter->15q11.2::20q10->20q11.1::20q13.33 ->20q13.33::11q23.2 ->11qter).
- r(20)(::20p11.1q11.21::19p13.?->19p13.?::).

The pattern and distribution of 20q11.2 segments in the abnormal chromosomes (Table S2, Fig. 1) was consistent with their being derived from a dicentric chromosome with an inverted duplication which included the region from the 20 centromere to 20q11.23 as proposed in Fig. 5, by BFB-induced rearrangement. Isodicentric chromosomes containing an inverted repeat of this region similar to the proposed precursor dicentric chromosome have been reported [31, 32].

These rearrangements included further duplication of the 20q11.2 inverted duplication that was separated from the 20 centromere-containing segments, to form the homogeneously staining region (hsr) in the der(9). A small acentric section of 20q11.22 between the sections that were represented in the r(20) and the hsr of the der(9) had been lost.

The composition of the der(19) and der(4;20) is consistent with their having arisen by duplication and subsequent evolution of the der(20) on two separate occasions, as illustrated in Figs 4A and 5. The der(20), the der(4;20) and the der(19) all contained a section of 11q carrying the *MLL* oncogene (Figs 1 and 5, Table S1). The der(19), der(4;20) and der(20) also contained the 20q11.1 paracentromeric material that was not represented on the SNP array, but of these only the der(4;20) and der(20) contained the 20 centromere. In the der(19), a 19 centromere in a 3 Mb section of chromosome 19 had replaced the 20 centromere (Figs 1 and 3B, Table S4). The der(20) and the der(19) also both contained the distal end of 15q (Fig. 4A). In specimens with the der(4;20), including our specimen, there were five copies of *MLL*, representing net gain of two copies from a triploid karyotype.

Amplification of 20q11.21 has been reported in erythroleukaemia with 20q12 deletion [33], notably the same disease that was present in the patient from which the HEL cell line was derived. Mechanisms of amplification in these patients have included formation of a ring chromosome 20 by excision of pericentromeric material from dicentric chromosomes [33, 34]. The ring 20 in HEL was apparently formed by a similar mechanism, and the region of greatest amplification reported by MacKinnon *et al.* [33] was present in both the der(9) and the excised ring 20 (Table S2). *E2F1* amplification has been reported in the hsr of the der(9) of HEL [35], but *E2F1* (which lies between RP11-120F10 and RP11-541L2) was not present in the ring 20 (Table S2).

The ring 20 was described in some early reports [2, 5, 7] and in the karyotype of the specimen held at the DSMZ cell line repository, which was the specimen karyotyped by MacLeod *et al.* [3, 4]. An add(15p) and a ‘del(6q)’ described in other publications [4, 5] matched the der(20)t(11;15;20) and the der(19) morphologically.

### Cell line heritage and evolution

While SNP array data alone do not reveal the content and organization of individual abnormal chromosomes, because our SNP array data correspond to detail on chromosome organization, we can draw conclusions about some of the abnormal chromosomes in other HEL sublines for which there are SNP array data available (two sublines in the CCLE [1] and one at the CGP [6]). Most of the CNAs were the same across all sublines. In Table 1, we list the major differences that were discernible by comparing SNP array data.

**Table 1.** A comparison of the SNP array patterns for the four different specimens of HEL, based on Fig. S1

Chromosome (copy number deviation in VCCS)	Abnormal chromosomes identified by M-FISH	Chromosomes abnormalities that match this SNP array pattern in the VCCS specimen		
	VCCS	DSMZ (CCLE)	ATCC (CCLE)	CGP
3 (loss)	der(3;18)	—^a^	der(3;18)	der(3;18)^a^^,b^
	der(6)t(3;6)		der(6)t(3;6)	der(6)t(3;6)
4 (loss, LOH)	der(4;20)	der(4;20)	der(4;20)	
4 (loss, LOH)	der(8)t(4;8)	der(8)t(4;8)	der(8)t(4;8)	
4 (loss)	der(4)t(4;14)			der(4)t(4;14)^a^^,b^
4 (gain)	Unknown			
5 (no loss; LOH)				3 copies 5qter—gain from normal homologue
8 (gain)	der(8)t(4;8;13)			
8 (gain)	der(8)t(4;8)	der(8)t(4;8)	der(8)t(4;8)	
10 (gain)	der(10;19) second copy	—^b^		der(10;19) second copy^b^
11 (loss, LOH)	der(9) breakpoint 11q22 with amplification at breakpoint	der(9) breakpoint 11q23	der(9) breakpoint 11q23	der(9) breakpoint 11q23
11 (gain)	der(4;20)	der(4;20)	der(4;20)	
13 (gain)	der(8)t(4;8;13)			
14 (gain)	der(4)t(4;14)			—^b^
15 (gain)	der(1)t(1;15)			
17 (no gain)				17qter gain from normal homologue
18 (loss)	der(3;18)		der(3;18)	
18 (loss, LOH)	r(18)			
18 (loss, LOH)	18qter on der(9)	Unknown telomeric segment on der(9)	Unknown telomeric segment on der(9)	Unknown telomeric segment on der(9)
19 (gain)	der(10;19) second copy	der(10;19) second copy^b^		—^b^
19 (gain)	der(19)	—^a^^,b^	der(19)	der(19)
20 (gain)	der(4;20)	der(4;20)	der(4;20)	
22 (loss)	−22	−22	−22	

**Figure 7.**
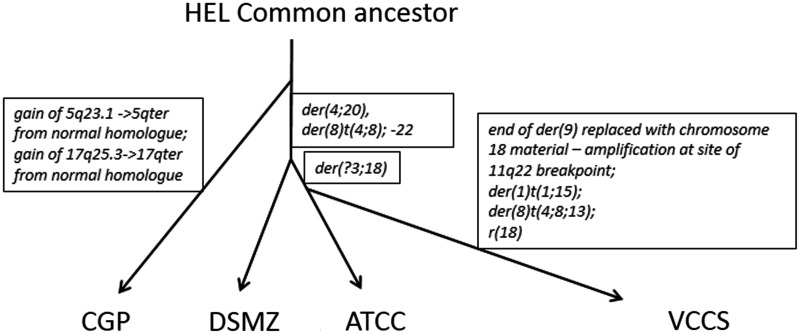
The suggested evolutionary pathway of the four sublines of HEL (VCCS represents this laboratory), as inferred by shared and disparate copy number abnormalities summarized in Table 1 and Fig. S1 The major differences in each branch are listed in the boxes. See text for caveats. Not to scale

The abnormal chromosome is listed in the VCCS column. In the DSMZ, ATCC and CGP columns, the abnormal chromosome is listed if SNP array data match the pattern for the VCCS specimen. ^a^This conclusion conflicts with the proposed evolutionary pathway (Fig. 7) or significance unknown. ^b^Data for both translocation partners conflict with this conclusion. Note that balanced translocations are not taken into account.

The 5;17 translocation is a recurrent unbalanced translocation [16] with net loss encompassing the CDRs 17p13 and 5q31.2, and is variously described as dic(5;17), der(5;17)t(5;17), der(5)t(5;17) or der(17)t(5;17) [16, 36, 37]. The unbalanced der(5;17), der(6)t(1;6), abnormalities of chromosomes 9 and 22 and the dup(21q) are distinctive abnormalities that were described in the earliest and all subsequent detailed HEL karyotypes. Published SNP array data match our SNP array data for these chromosome abnormalities, consistent with their presence in the CGP, DSMZ and ATCC lines [3, 4, 38].

The rearrangements producing the der(2)t(2;10), der(10)t(2;10;18) and der(18)t(10;18) were balanced in our specimen, i.e. there was no net CNA of the involved chromosome 2, 10 and 18 homologues (see Fig. 1). Greenberg *et al.* described a der(10)t(2;10)(10;?) and a der(2)t(2;10) matching the morphology of our der(10)t(2;10;18) and der(2)t(2;10) in the HEL-contaminated Dami specimen in 1988 [5]. Neither is there CNA of these chromosome 2, 10 and 18 homologues in the three specimens with online SNP array data (DSMZ and ATCC [1], and CGP [6]), consistent with these balanced rearrangements being present in these specimens and having arisen before divergence of the three different HEL cell line stocks.

The complex SNP array patterns produced by the chromosome 9, 15, 19p and 20 aberrations were largely consistent with the CGP and CCLE specimen SNP array data, supporting an early origin for the der(9), psu dic(22;9), der(20), der(19) and r(20). SNP array data showed that the der(4;20) was present in the ATCC and DSMZ specimen but not the CGP specimen. This is evidenced by the absence of a fifth copy of 20q13.33, of a fifth copy of the *MLL*-containing chromosome 11 region, and of the associated chromosome 4 deletion in the CGP specimen. The ATTC and DSMZ SNP array patterns matched SNP array patterns associated with both the der(4;20) and the der(8)t(4;8), which were derived from the same chromosome 4 homologue.

Shared patterns of LOH should be a good indicator of a common ancestry, as heterozygosity cannot be regained. LOH of chromosome 4 in our specimen and the DSMZ and ATCC specimens suggests that they belong to a common lineage that is more distant from the CGP specimen. Lack of the der(4;20) in the CGP specimen is consistent with this derivative having arisen after the der(20)t(11;15;20) (Fig. 4A). Using the comparison in Fig. S1 and Table 1, we propose the evolutionary tree in Fig. 7. There are some uncertainties and inconsistencies in the SNP array data (see Table 1), possibly because of uncovered balanced rearrangements in the CCLE and CGP SNP array data or the same minor subclones becoming predominant in different sublines. These uncertainties could probably be resolved using whole chromosome methods such as we used here (e.g. M-FISH).

We suggest that the VCCS specimen is closest to the ATCC specimen [1], as both had the same CNAs associated with the der(?3;18) (3p and 18p deletion), which were lacking in the DSMZ specimen (Fig. 7, Table 1), although this is not clear-cut. The excess of chromosome aberrations in the VCCS specimen probably reflects its continued culture compared with the parent stock. Cells in our specimen had either the der(8)t(4;8;13) or der(8)t(4;8), an example of evolution in progress.

While chromosome 9 and 20 aberrations were largely identical across all the specimens, chromosome 11 and 18 SNP array data [1, 6] revealed that the capping of the der(9) with chromosome 18 material was unique to our specimen. The distal part of 11q and the unknown telomere-containing segment in the der(9) of the CGP and CCLE specimens had been replaced with the terminal end of 18q in the VCCS specimen (Fig. 4B). There was also gain of two copies of a short section of 11q at the 11q site of breakage on the der(9) in our specimen (Figs 1 and 3C). A separate section of this rearranged chromosome 18 homologue formed the ring 18 (Fig. 1). Of the four specimens analysed by SNP array, these rearrangements were only detected in our specimen.

Reports of a del(20q) in HEL by traditional karyotyping [4, 5, 7] appear to be inaccurate, particularly in light of SNP array data in the four sublines which suggest that most chromosome 20 aberrations were present when the cell line was established (see above). MacGrogan *et al.* [7] identified three chromosomes that were positive for the 20 centromere and negative for *D20S108*, the morphology of which matched our three chromosome 20 derivatives (containing the 20 centromere). The chromosome identified as a del(20q) by MacGrogan *et al.* [7] on unbanded chromosomes matches the sub-metacentric der(4;20)t(4;20;11), in shape and relative size. Both are positive for the 20 centromere and negative for *D20S108*. It seems likely that the der(4;20) was therefore misclassified as a del(20q) by MacGrogan *et al.* [7].

Some material distal to the 20q12 deletion was retained, a pattern that is common in unbalanced 20q translocations [39]. Deletion of 20q12 is often an early chromosome aberration in more complex karyotypes [40] and it is possible that a del(20q) preceded the complex chromosome 20 abnormalities that were found in HEL. Alternatively, fusion between the 20q telomere and another chromosome may have led to 20q deletion via the BFB cycle [39].

The same chromosome 6 homologue that was involved in the der(19)t(6;11;15;19;20) was involved in the der(6)t(3;6), and no net gain or loss of this chromosome 6 homologue is shown by our SNP array data or the published SNP array data. This is consistent with the der(6) and the der(19), and by extrapolation the der(20), being early aberrations.

Other conclusions about the sequence of events during evolution of the HEL genome could be drawn from BAFs. In the triploid karyotype, if one copy of the duplicated homologue was rearranged, this must have occurred after the doubling of this homologue. These abnormalities included the der(6)t(3;6), der(?3;18)t(3;18), der(4)t(4;14), t(2;10;18) and the der(19) (because it involved the same chromosome 6 homologue as that from which the der(6)t(3;6) was derived).

### Centromere and telomere capture

Telomere capture has been described as a mechanism helping stabilize rearranged chromosomes [41]. We identified rearrangements consistent with telomere capture, as well as the harnessing of short chromosome sections containing centromeres, to help form stable chromosomes.

Three abnormal chromosomes derived from the der(20) (the der(19), der(20) and der(4;20)) mostly comprised material from other chromosomes. In particular, the inclusion of the 20 centromere and/or paracentromeric region with little surrounding material in these chromosomes is striking. We suggest that the capture of a short segment containing a centromere on at least two occasions (creating the der(19) and der(20)) has allowed a duplicated *MLL*-containing segment to be preserved. A proposed evolutionary history of these derivatives suggested by their shared chromosome 11, 15 and 20 components is illustrated in Fig. 4A.

The der(19) was an early aberration that appears to have been derived from a broken der(20), as it contains a subset of the chromosome 11, 15 and 20 material from the der(20) as discussed above (Fig. 4A) and the remainder of the chromosome 6 material that is present in the der(3)t(3;6). The captured segment containing the 20 centromere in the der(20) and duplicated in the der(4;20) appears to have been produced as a by-product of the BFB cycle (see above; Fig. 5), and the presence of other parts of these chromosome 11 and 20 homologues in the der(9) suggests that there were simultaneous breakage events involving these chromosomes.

We recently reported the use of a chromosome segment containing a centromere in the remodelling of chromosomes after chromothripsis in two cases of AML [42]. Together with evidence from HEL, these examples point to a previously undescribed mechanism for stabilizing chromosomes: centromere capture. To our knowledge, these are the first descriptions of centromere capture. The centromere content of abnormal chromosomes is best determined by FISH, and is not usually investigated. Neocentromeres, which are centromeres created *de novo* by chromatin remodelling without underlying satellite DNA [43], can form on abnormal chromosomes that contain oncogenes but no native centromere [44, 45]. Both of these mechanisms could be used to stabilize broken acentric chromosome segments and prevent them from being lost. If the broken segment contained an oncogene, acquisition of a functional centromere and telomeres could help preserve it and provide a selective advantage to the cell.

Yu and Graf [46] showed that telomere capture can be used to stabilize a terminal chromosome deletion associated with an inverted duplication. The use of the reduced telomeric section of 15q from the der(20) to cap the inverted duplication of 11q in the der(19) follows this pattern (Fig. 4A).

## Conclusions and implications

With the growing capacity to sequence cancer genomes, genome organization, particularly the difficult-to-sequence repetitive DNA regions such as telomeres and centromeres are often overlooked. Yet, these regions are undoubtedly important to chromosome stability and cancer evolution. As demonstrated here, the content and organization of different rearranged chromosomes can give clues to their origin. This study highlights the variety of complementary methods that are required to understand remodelling of the genome in cancer cells, particularly methods which pay due attention to repetitive sequences, which can play a crucial role in chromosome stability and rearrangement.

The combined use of several different molecular cytogenetic approaches can be used to help determine the organization of a complex karyotype. A useful feature of the BAF pattern is that it can help distinguish between the aberrations occurring on two different homologues. The availability of SNP array data and corresponding karyotype data allows the karyotypes of other sublines to be largely deduced from SNP array data alone.

Of the chromosome 20 abnormalities, the data were consistent with a der(9), the r(20), der(19) and der(20) being early abnormalities in the cell line. The der(4;20) appears to have been derived from the der(20) in the DSMZ/ATCC specimens after separation from the CGP specimen by a rearrangement involving whole arm translocation of 4p. The original der(9) had undergone subsequent rearrangement in our specimen, with an 18q telomeric segment replacing the original (unknown) telomeric segment. Many of these details could not have been determined from microarray analysis alone without information on the structure of the abnormal chromosomes.

Metaphase FISH is a powerful technique for helping to determine the content and organization of abnormal chromosomes. For highly repetitive sequences, there is no viable alternative. FISH helped identify the apparent capture of centromeres to stabilize potentially oncogenic chromosome segments. FISH for another repetitive DNA element, the nucleolus organizer region, revealed the likely sequence of events that produced the chromosome 9-containing derivative chromosomes. The identification of translocation partners and the location of rearranged chromosome regions were made possible with M-FISH, M-BAND and single locus FISH.

A comparison of our karyotypes with previously published conventional and SNP array karyotypes for this cell line showed that although there were some abnormalities in common, there were other rearrangements which were not shared, illustrating the continuing evolution of this cell line in culture. The diversity of published karyotypes of this cell line highlights the ongoing adaptive evolution of the genome during continued culture and passage. One or more of the four HEL sublines did not have the same level of gain of *MLL*, *TET2* deletion and LOH or chromosome 18 deletion, known leukaemogenic aberrations that were present in other sublines. The changing genome should be considered when cell lines are used to study cell and cancer biology.

Mechanisms of CNA in cancer cells and cell lines are still not well understood. This cell line shows how the BFB cycle can contribute to chromosome instability, creating both amplifications and deletions which provide material for selective evolution.

This type of detailed analysis will be useful in determining the steps that produce highly abnormal genomes, both *in vivo* (cancer) and *in vitro* (cell lines). Complex genome reorganization is a feature of many cancers, often contributing to malignancy, but is difficult to analyse. Cytogenomic arrays give copy number information but do not determine the structure of the abnormal chromosomes. The combination of molecular cytogenetic approaches used here illustrates a powerful approach to studying the structure and evolution of a complex abnormal genome.

## Supplementary Material

Supplementary Data
