# Supplementary material for: Genome organization and the role of centromeres in evolution of the erythroleukaemia cell line HEL
Source: Evol Med Public Health. 2013 Oct 1;2013(1):225–40. doi: 10.1093/emph/eot020 (PMC3868402; doi:10.1093/emph/eot020)
Supplement: Supplementary Data [file supp_2013_1_225__index.html]

Genome organisation and the role of centromeres in evolution of the erythroleukaemia cell line HEL — Genome organization and the role of centromeres in evolution of the erythroleukaemia cell line HEL — Supplementary Data 

# Genome organization and the role of centromeres in evolution of the erythroleukaemia cell line HEL

## Supplementary Data

files

**Files in this Data Supplement:**

- Supplementary Data - docx file
- Supplementary Data - tif file
- Supplementary Data - xls file
- Supplementary Data - xls file
- Supplementary Data - xlsx file
- Supplementary Data - xlsx file
