# Supplementary material for: Genome organization and the role of centromeres in evolution of the erythroleukaemia cell line HEL
Source: Evol Med Public Health. 2013 Oct 1;2013(1):225–40. doi: 10.1093/emph/eot020 (PMC3868402; doi:10.1093/emph/eot020)
Supplement: Supplementary Data [file supp_eot020_Supplementary_Figure_1_caption.docx]

Supplementary Figure 1. VCCS HEL SNP array idiograms from Figure 1 showing a comparison with DSMZ (CCLE), ATCC (CCLE) and CGP SNP array patterns. A blue bar represents loss compared to the VCCS specimen and a red bar represents gain compared to the VCCS specimen.
