# Supplementary figures and images for: Genome organization and the role of centromeres in evolution of the erythroleukaemia cell line HEL
Source: Evol Med Public Health. 2013 Oct 1;2013(1):225–40. doi: 10.1093/emph/eot020 (PMC3868402; doi:10.1093/emph/eot020)

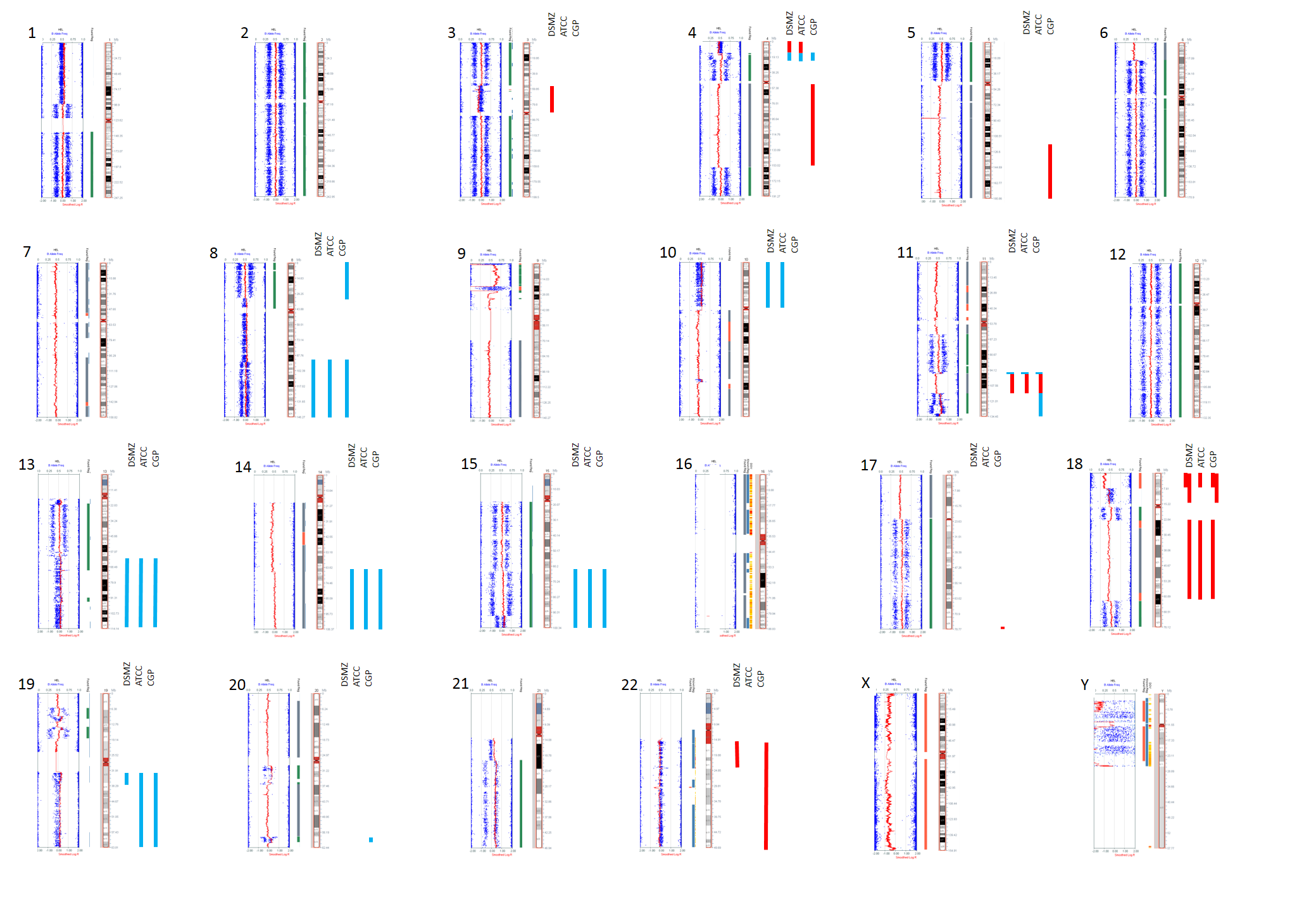

Supplement: Supplementary Data [file supp_eot020_supp_figure_1_compare_snp_data.tif]
